# Beyond “Non-Significant” Results: Why and How to Test for Practical Equivalence

**DOI:** 10.1073/pnas.2611548123

**Published:** 2026-08-14

**Authors:** Jakub Tomek, Aaron Caldwell, David Eisner

**Affiliations:** 1 Department of Anatomy, Physiology, and Genetics, University of Oxford; 2 Department of Biostatistics, Fay W. Boozman College of Public Health, University of Arkansas for Medical Sciences Northwest, Springdale, AR, USA; 3 Division of Cardiovascular Sciences, University of Manchester

## Abstract

Reporting comparisons with p ≥ 0.05 as showing “no effect” or “no difference” remains one of the most widespread and problematic misinterpretations in the scientific literature. A statistically non-significant result shows only that the data do not provide strong evidence for a difference. This distinction matters because such findings can arise for two very different reasons: either there is no meaningful difference, or a meaningful difference is present but cannot be detected reliably because of limited sample size or high variability. We highlight equivalence testing as a practical framework for distinguishing between these possibilities. Using the two one-sided tests (TOST) approach, investigators can formally test whether effects large enough to be scientifically, clinically, or practically meaningful can be ruled out. We explain the logic of TOST, show how it distinguishes practical equivalence from inconclusive evidence, and illustrate its use in paired and unpaired comparisons. To support broader adoption, we also introduce a freely available online calculator that enables researchers to perform common equivalence tests without coding.

## Article

A persistent challenge across the life and natural sciences is how to interpret statistically “non-significant” results (e.g., p ≥ 0.05). Such findings are routinely summarized as “no difference” or “no effect,” despite long-standing recognition that this is a fundamental misinterpretation (1–4). A non-significant p-value indicates merely an absence of evidence for a difference, not evidence that no difference exists. While a true absence of an effect may indeed produce a non-significant result, the same outcome can arise when an effect is real but difficult to detect with the given number of subjects or data variability.

The continued prevalence of this error in publications and conference presentations suggests that repeated warnings alone are insufficient (3, 5). One reason may be that researchers often face a genuine need to demonstrate that an effect is absent or negligible. Unfortunately, mainstream statistical testing cannot show that a difference is exactly zero. However, it does allow researchers to address a closely related and often more meaningful question: whether any difference that exists is too small to matter for practical purposes (“practically irrelevant”).

*Equivalence testing* provides a formal statistical framework for answering this question. Specifically, it tests whether the observed effects are sufficiently small to be considered negligible. Importantly, equivalence testing relies on familiar tools such as t-tests, making it accessible to researchers already trained in standard statistical methods. The intuition behind equivalence testing is straightforward. When observed differences are tightly clustered around zero across many observations, it becomes increasingly implausible that a large or meaningful effect is present. Conversely, when there are few measurements and/or they are highly variable, a lack of statistical significance absolutely should not be taken as evidence of practical equivalence. Equivalence testing formalizes this distinction and helps separate genuinely small effects from inconclusive data.

Although equivalence testing has been increasingly adopted in psychology following clear methodological guidance (6, 7), and has a long history in clinical, pharmaceutical, and regulatory contexts (8–10), it remains underused in the life and natural sciences. This is surprising because equivalence testing answers precisely the question a non-significant result cannot: is the effect too small to matter, or does the evidence remain inconclusive? In this Perspective, we outline the principles of equivalence testing and introduce a freely available online tool designed to facilitate its broader use.

### The logic of equivalence testing

A widely used approach to equivalence testing is the two one-sided tests (TOST) procedure(6). The first step is to define a range of effect sizes considered too small to be of practical relevance. These lower (ΔL) and upper (ΔU) equivalence bounds define the interval within which effects are treated as negligible. One could e.g. say that if a treatment changes a given protein’s expression by less than 5% in either direction, it is too small an effect to be of importance.

The procedure then tests two null hypotheses: that the true difference is more positive than ΔU, or more negative than ΔL. If both hypotheses are rejected at a chosen significance level (typically 0.05), one can conclude statistical equivalence – i.e., the effect is unlikely to be large enough to matter in either direction. Effectively, we try to assume that there is a large-enough difference in one or the other direction, and if we falsify both assumptions, we can conclude that such a large-enough difference is absent.

### Interpreting non-significant results in practice

**Figure 1:**
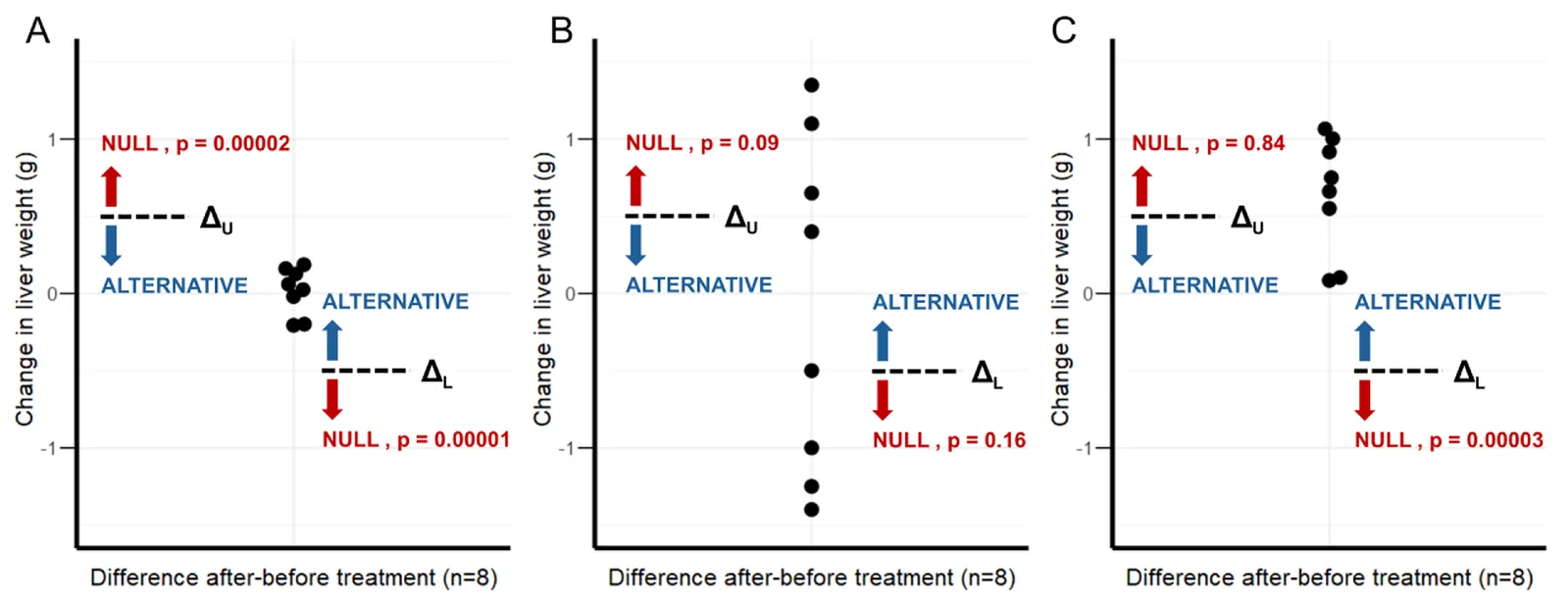
Equivalence testing for paired comparisons using the two one-sided tests (TOST) procedure. The paired comparisons show changes in liver weight following an intervention, with equivalence bounds set at ±0.5 g. In **(A)**, differences are tightly clustered around zero and fall within the equivalence bounds; both one-sided null hypotheses are rejected, supporting statistical equivalence. In **(B)**, the data are much more variable, and it does not hold that both p-values are <0.05; therefore, equivalence within the equivalence bounds cannot be concluded. In **(C)**, data are not tightly clustered around zero and only one null hypothesis is rejected, so equivalence cannot be concluded. One-sided, one-sample t-tests were used for TOST.

Figure 1 illustrates the use of equivalence testing in the setting of paired comparisons. There, equivalence testing assesses whether within-subject differences are sufficiently small to be considered practically irrelevant. When observed changes are tightly clustered around zero and fall well within the equivalence bounds, both one-sided tests reject their null hypotheses, supporting a conclusion of practical irrelevance (Figure 1A). When variability is larger (Figure 1B) and/or the data are not clustered around zero (Figure 1C), equivalence cannot be established.

**Figure 2:**
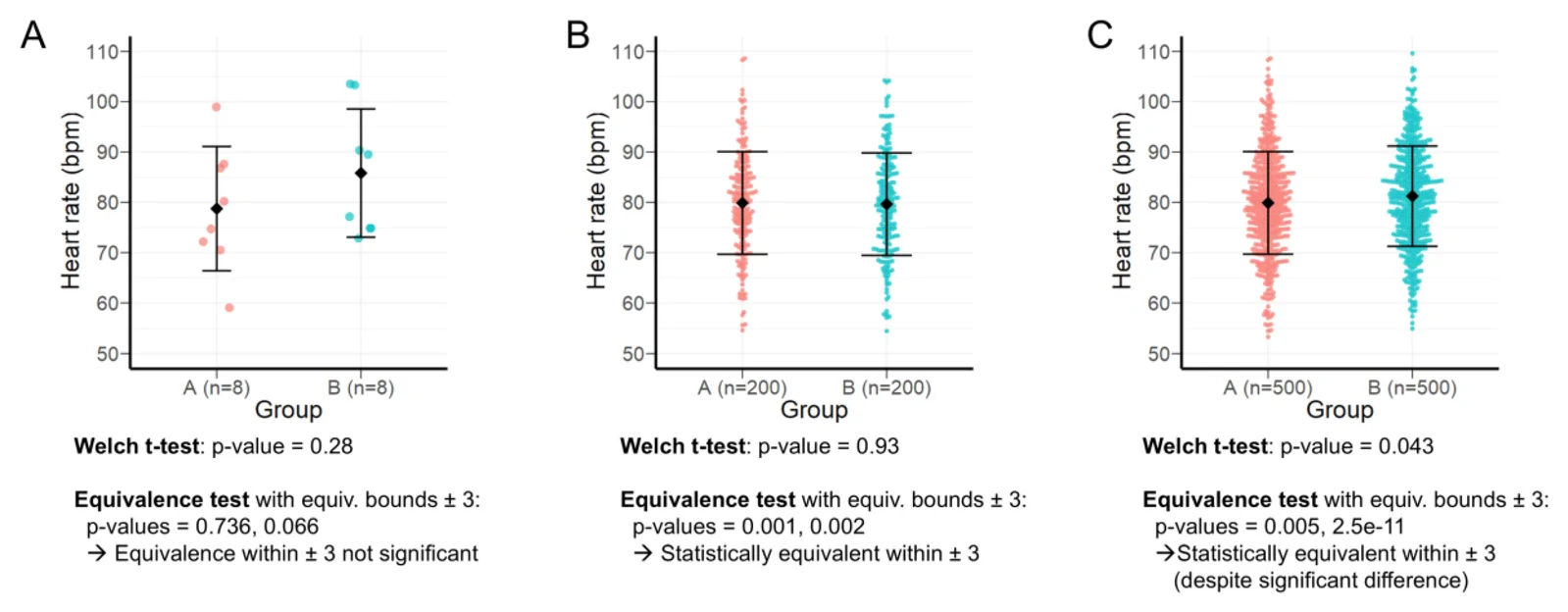
Examples of equivalence testing for unpaired comparisons. Shown here are unpaired comparisons of heart rate between two groups. Equivalence bounds are chosen at ±3 beats per minute (bpm). In **(A)**, neither the test of difference nor the test of equivalence is significant, indicating inconclusive data. In **(B)**, the difference is non-significant, but both equivalence tests are significant, supporting practical equivalence. In **(C)**, both the test of difference and the test of equivalence are significant, illustrating a small but statistically detectable difference that is nevertheless practically irrelevant. Welch’s t-test was used for tests of difference, and TOST based on one-sided Welch tests is used for equivalence testing. This follows recommendation of Welch t-test as a suitable default approach, rather than historically more common pooled-variance t-test (11, 12).

Equivalence testing can also be easily used when there are two unpaired groups compared using e.g. a t-test. In Figure 2A, a comparison of heart rate is made between two small groups of subjects, with p = 0.28 for the difference using the Welch t-test. It is unfortunately very easy to imagine an article incorrectly reporting this comparison as “Heart rate did not differ between groups (p>0.05)”. If a difference of up to ±3 beats per minute (bpm) is deemed practically irrelevant, one of the one-sided p-values is high, not supporting the claim of equivalence. In this case, the data are generally inconclusive, supporting neither a statistically significant difference nor statistical equivalence within ±3 beats.

An example of statistical equivalence accompanying non-significant difference is shown in Figure 2B. With many measurements underlying the apparent lack of substantial difference, both TOST p-values fall below 0.05. Under the same equivalence bounds ± 3 bpm, the groups can now be considered statistically equivalent. These two cases illustrate how equivalence testing distinguishes between general lack of evidence and evidence of practical irrelevance.

In some situations, both a test of difference and a test of equivalence may be statistically significant (Figure 2C). This occurs when very small effects are detected with high precision in large samples. Such results highlight a critical distinction: statistical significance is not the same as practical or clinical significance. Equivalence testing makes this distinction explicit by allowing researchers to conclude that a detected difference, while real, may be too small to matter.

### Choosing equivalence bounds

A crucial step in equivalence testing is defining the smallest effect size of interest (SESOI) (6, 13, 14). The SESOI specifies the threshold below which effects are considered practically or theoretically negligible. In other fields and contexts, alternative terms are used to refer to the same concept, such as Minimum Important Difference (MID) or Minimum Clinically Important Difference (MCID) (15, 16). There is no universal rule for choosing these bounds; they depend on the research context, our knowledge of the system, and the consequences of acting on a given result.

Useful guiding questions include:

- “What is the smallest difference that still supports my theory?”
- “What size effect would make me pursue research on this topic in greater depth?”
- “Is there a standard range in my field?”, In pharmacokinetics, for instance, bioequivalence is established when the 90% confidence interval for the geometric mean ratio of test to reference drug falls within 0.80–1.25 (17).
- “What is the smallest result that would lead to a change in clinical practice?”
- “What result would make us try to develop a compound into a drug?”

While answers may vary among researchers, this subjectivity is not a flaw. Scientific inference always needs to consider context. Explicitly articulating and justifying a SESOI makes the consideration of context underlying an equivalence test transparent and open to scrutiny.

To give a concrete example, imagine a study of skeletal muscle fatigue following a drug treatment, which, when given at a high concentration, reduces forces to 50% of the control value. This might in principle result from muscle acidification. Based on prior work, an acidification of approximately 0. 3 pH units might produce this decrease of force in comparable conditions and muscle type. If the intracellular pH can be measured in the current study, the SESOI can be defined relative to this expected change. For instance, the equivalence bounds could be set conservatively at ±0.1 pH units versus untreated muscle. If the observed change in pH falls within those equivalence bounds, this would argue against intracellular acidification as a sufficient explanation for the observed fatigue, and a different mechanism would have to be sought.

### Using confidence intervals

Equivalence can also be assessed through confidence intervals. For a TOST procedure conducted at α = 0.05, equivalence is concluded when the corresponding 90% confidence interval for the effect lies entirely within the prespecified equivalence bounds (6). Because that interval is constructed as the pair of the two one-sided tests, the confidence interval inclusion rule and the equivalence test criterion *p* < 0.05 are not competing approaches but a single decision expressed two ways. Beyond that shared decision, however, the two summaries convey complementary information. The confidence interval reports the range of effect sizes compatible with the data, whereas the exact *p*-value, as opposed to the bare *p* < 0.05, gives a continuous measure of the discrepancy between the data and the null hypothesis, one that can be read either as graded evidence or as the basis for a formal decision (18).

### Software for equivalence testing

Equivalence testing via the TOST procedure is currently most easily done via the TOSTER package in R (6, 19). To promote broader adoption, particularly among researchers who do not wish to write code, we have developed a freely available user-friendly online calculator (https://teacup-equivtest.streamlit.app/), validated against TOSTER.

The online calculator supports equivalence testing for common parametric and nonparametric comparisons, including a range of t-tests (one-sample/paired, two-sample, Welch’s two-sample), nonparametric tests of stochastic superiority (two-sample and paired Brunner-Munzel), and Wald-type test of independent proportions test for contingency tables. This coverage allows equivalence testing to be applied to many standard comparisons of two samples, paired samples, or contingency tables. There are plans for additional tests to be added in future versions.

### Implications for scientific inference

By lowering technical barriers to equivalence testing, we hope to reduce the widespread misinterpretation of non-significant results. Considering equivalence alongside conventional tests of difference enables more nuanced conclusions: an effect may be present and meaningful, present but negligible, or simply unresolved by the data. In order for equivalence testing to be informative and improve statistical reasoning, we strongly recommend that authors state the equivalence bounds prominently when reporting equivalence results and justify their choice.

Finally, while we have framed equivalence testing using conventional significance thresholds for clarity, this is not the only framework of statistical inference. Binary decision rules (e.g., “significance was set at *p* < 0.05”) can be useful when formal error control is required, such as in clinical, regulatory, or pre-registered settings, but may be unnecessary or inappropriate in some settings, e.g., exploratory work (3, 20). As the interpretation of *p*-values shifts toward a continuous scale, TOST *p*-values can be read the same way, with equivalence increasingly supported as both one-sided *p*-values decrease (3, 21). Alternatively, the 90% confidence interval can be compared directly with the equivalence bounds. Whatever the approach, effect sizes should always be reported, ideally alongside a measure of uncertainty.

Recognizing the difference between “no evidence of a difference” and “evidence of no meaningful difference” can substantially improve how scientific results are interpreted, reported, and acted upon.
